# Impact of HIF1-α/TGF-β/Smad-2/Bax/Bcl2 pathways on cobalt chloride-induced cardiac and hepatorenal dysfunction

**DOI:** 10.2144/fsoa-2023-0050

**Published:** 2023-07-13

**Authors:** Mai O Kadry, Hanaa Mahmoud Ali

**Affiliations:** 1 Therapeutic Chemistry Department, National Research Centre, El Buhouth St., Dokki, 12622, Egypt; 2 Department of Genetics & Cytology, National Research Centre, El Buhouth St., Dokki, 12622, Egypt

**Keywords:** AKT, Bax/Bcl2 ratio, cobalt chloride, HIF1-α, Smad-2, TGF-β

## Abstract

**Background:**

Cobalt chloride (CoCl_2_) is a ferromagnetic ubiquitous trace element extensively dispersed in the environment. Nevertheless, it may merit human hazard.

**Aim:**

Excess cobalt can harm vital organs this paves the way to elucidate the toxic impact of CoCl_2_ on the liver, kidney and heart.

**Method:**

CoCl_2_ was injected in a dose of (60 mg/kg, S.C.) proceeded via Carnosine (200 mg/kg) and/or Arginine (200 mg/kg) treatment 1 month, 24 and 1 h, prior to CoCl_2_-intoxication.

**Results:**

CoCl_2_ significantly alleviated hemoglobin concentration and BCl2; meanwhile, protein expression of transforming growth factor (TGF-β), hypoxia-inducible factor (HIF-1α), Mothers against decapentaplegic (Smad-2), AKT protein expression and Bax/Bcl2 ratio was noticeably elevated.

**Conclusion:**

The combination of the aforementioned antioxidants exerted a synergistic anti-apoptotic impact in all target tissues.

## Plain language summary

Cobalt chloride (CoCl_2_) is commonly found in the environment and used in medicine. However, it can be harmful to our health, particularly when consumed in excessive amounts, leading to damage in important organs. Therefore, we investigated the toxic effects of CoCl_2_ on the liver, kidney, and heart. We also explored potential treatments using substances like Carnosine and Arginine. We discovered that Arginine and carnosine had a positive effect on certain factors related to the health of the organs. They helped regulate the levels of hemoglobin and BCl2, as well as the expression of proteins such as transforming growth factor (TGF-β), hypoxia-inducible factor (HIF-1α), Mothers against decapentaplegic (Smad-2), AKT, and apoptotic biomarkers like the Bax/Bcl2 ratio. When these antioxidants were combined, they had a stronger protective effect against cell death and mutations in all the organs studied.

The 20th century showed a tremendous spread in cobalt utilization, which revealed a substantial impact on atmospheric, aquatic and terrestrial habitats. It is essential for enzyme activation, vitamin B_12_ biosynthesis and other biological processes, but toxic in high concentrations. CoCl_2_ is a blood doping agent in both people and animals, along with its medical significance in Co-containing hip implants for patients undergoing surgery [1]. Nevertheless, it is among the most hazardous environmental pollutants, that affects hepatic, renal, hemopoietic and nervous systems thus its human exposure is overly concerning [2]. CoCl_2_ has been linked to harmful side effects including thyroid malfunction in children and many cardiovascular diseases [3]. CoCl_2_-toxicity has previously been linked to hepatotoxicity, nephrotoxicity, cardiotoxicity as well as hypotensive and hypertensive effects at various dosages and experimental setups. Similarly, a noticeable connection between lowered serum nitric oxide and CoCl_2_ intoxication was discovered. Additionally, it inhibited the hypoxia-inducible factor (HIF-1α), which led to cytotoxicity and inflammation [1,4–6]. Additionally, CoCl_2_ prompted caspase-3 and caspase-6 activation, DNA fragmentation and apoptosis in rat and mice models [7–9].

The toxicity of CoCl_2_ has been linked to oxidative stress, inflammation, and apoptosis. Oxidative stress is an imbalance between reactive oxygen species (ROS) and antioxidant defense mechanisms. The Fenton reaction and the subsequent induction of ROS by cobalt ions can lead to the oxidative alteration of cellular lipids, proteins and DNA. An earlier study revealed the potential hepatic harm caused by CoCl_2_ misuse, as well as the toxicological effects on the liver's antioxidant status, apoptosis, genotoxicity and molecular mode of action [9].

Genotoxicity of cobalt compounds was confirmed by ECHA, 2017 and Kirkland *et al.*, 2015 [10,11] who reported cobalt chromosomal aberrations, micronuclei and DNA damage *in vitro*. Moreover, it produced aneuploidy, oxidative DNA-damage, and micronuclei, in *in vivo* studies [10,12] It is thought that this genotoxicity is mediated via secondary mechanisms that is related to ROS induction and initiation of oxidative stress [10,13,14].

CoCl_2_ significantly increased the production of ROS, phosphorylated p38 mitogen-activated protein kinase (MAPK), and damaged neurons [15]. It has been suggested that the ROS produced by cobalt chloride have a role in the signaling mechanism that stabilizes the transcription factor HIF-1α during hypoxia.

The epithelial-mesenchymal transition (EMT) takes place during adult tissue remodeling processes such as fibrosis and carcinogenesis. Hepatocytes can undergo EMT in the adult liver, which is crucially involved in chronic liver injury, according to the evidence that is currently available. CoCl_2_ was reported to promote a mesenchymal cell phenotype in human LO2 hepatocytes and up-regulate many mesenchymal markers, including -smooth muscle actin and fibronectin, meanwhile down-regulated the epithelial marker E-cadherin [16].

Transforming growth factor (TGF-β) signaling was overexpressed by CoCl_2_, and Smad-2 and Smad-3 expression and phosphorylation were also increased [17].

CoCl_2_, a chemical chelator that replaces the ferrous+ ion in hemoglobin, causes harm to the cell's oxygen receptors [18]. In human cancer cells, CoCl_2_ is linked with both growth and apoptosis [19].

Studying the several methods by which natural products operated as anti-apoptotic agents offers the potential for the creation of novel medications. Natural products have the potential to be a source of hepatoprotective and renoprotective inhibiting apoptosis of all tissues [20].

Along with natural antioxidants, the product responsible for the synthesis of NO, which has various physiological functions including neurotransmission, vasodilation, vaso-relaxation, and immune system modulation, is arginine (Argi) [21]. Recent studies stated that Argi possesses both anti-inflammatory and antioxidant properties [22,23]. NO possesses anti-apoptotic potential at physiological levels and can modify the inflammatory response by lowering C-reactive protein (CRP) and inflammatory cytokines, which causes organ damage [24,25]. According to El Fakahany [26] (2021) and Soliman *et al.* (2023) [27], Argi enhances renal function impaired by Acrylamide and fipronil.

Carnosine (Carn) is a histidine dipeptide that is naturally occurring and endogenously generated in human body, carnosine is broadly distributed in the kidney, brain, muscle, stomach and in significant proportions, skeletal muscle [28]. Numerous biological actions of this dipeptide have been demonstrated, including the capacity to chelate metal ions, anti-oxidant activity, suppressing protein glycosylation, anti-inflammatory impact and anti-senescence properties. The anti-proliferative action of L-carnosine in human cancer cell lines is another impact [29,30]. Carnosine possesses anticancer properties both *in vivo* and *in vitro* via inhibiting proliferation of human colorectal carcinoma cells by affecting ROS, ATP production and arrest cell cycle at G1 phase. HIF-1α as a possible target of L-carnosine in HCT-116 cell line was previously examined. HIF-1α is over-expressed in various kinds of human cancers and is the main reason of radiation and drugs resistance in solid tumors. Carnosine limits HIF-1α transcriptional activity and lessens HIF-1α protein expression disturbing its stability. Carnosine is elaborated in ubiquitin-proteasome system enhancing HIF-1α degradation thus inhibiting tumorgenesis. It possesses cardioprotective properties in cardiomyoblasts [31].

The current study's objective is to track the underlying processes by which the natural antioxidants Argi and/or Carn act as anti-apoptotic and antimutagenic agents against toxicity caused by CoCl_2_ in various organs, including the liver, kidney and heart.

## Impact statement

Currently, there is a wide use of different metals in various medicinal fields. Cobalt chloride has been used as a blood doping agent in both people and animals, along with its medical significance in co-containing hip implants for patients undergoing surgery. However, it has been linked to thyroid malfunction in children and several cardiovascular diseases. Studying methods by which natural products act as anti-apoptotic and antimutagenic agents offers opportunities for the exploration of new pharmaceutical medications. Arginine and Carnosine possess a powerful anti-inflammatory and antioxidant impact. The aim of the current work is to find the underlying mechanisms that the natural antioxidants Arginine and/ or Carnosine can perform versus CoCl_2_ toxicity in different rat organs. Data showed that Haemoglobin concentration was dramatically reduced by CoCl_2_, although AKT, Smad-2, TGF-β, Bax/Bcl2 ratio, HIF-α were increased. The aforementioned antioxidants elucidated a synergistic anti-apoptotic and anti-mutagenic impact on all target organs which may be mediated via various growth factors signaling pathways.

## Materials & methods

### Chemicals

All chemicals used were of high analytical grade, the product of Sigma and Merck companies. Carn, Argi and CoCl_2_ were obtained from Sigma Chemical Co. (Sigma, MO, USA).

### Animals

Adult male albino Wister rats (200–220 g; 6–8 weeks old) were obtained from the Animal House at the National Research Centre. Animals were kept in special cages and maintained on a constant 12 h light/dark cycle with a controlled temperature of 20–22°C and humidity of 60%. Rats were fed a standard rat pellet chow with free access to tap water ad libitum for 1 week before the experiment for acclimatization. After one week of acclimation, the rats were kept fasting overnight before treatment and randomly divided into six groups, each of ten rats.

### Experimental design

Fifty rats were randomly divided into five equal groups ten rats each.

**Group I:** Control will be injected intraperitoneally with saline.

**Group II:** Rats were injected with a single dose of CoCl_2_ (60 mg/kg, s.c.) [32].

**Group III:** CoCl_2_- intoxicated rats pretreated with Carn for one month (200 mg/kg/day) [33].

**Group IV:** CoCl_2_- intoxicated rats pretreated with Argi for one month (200 mg/kg/day) [34].

**Group V:** CoCl_2_-intoxicated rats pretreated with Carn and Argi for one month.

Carn and Argi were orally administrated twice weekly for one month and (24 and 1 h) before CoCl_2_ intoxication; as preventive strategy to mitigate CoCl_2_ intoxication.

### Samples collection & preparation

After 6 h from CoCl_2_-intoxication, the experiment animals were fasted overnight and then sacrificed and blood samples were collected for Hb determination and serum analysis, liver, heart, and kidney tissues were collected thoroughly in ice-cold saline and were rapidly frozen in liquid nitrogen and stored at -80°C for Western blotting analysis.

## Methods

### Determination of hemoglobin (Hb)

Hb was determined colorimetrically in blood using Drabkin's reagent according to the method of Kjeldsberg [35].

### Assay of liver function

Serum transaminases (alanine transaminase; ALT and aspartate aminotransferase; AST) were assessed by Randox (Crumlin, UK) kits following the provided directions.

### Assay of kidney function

Urea, and creatinine in serum were estimated, according to the instructions of the manufacturer. Diagnostic kits were bought from Randox Company Chemical CO.

### Assay of heart function

Troponin T (Trop. T) concentration was evaluated using a Siemens Dimension Xpand Plus instrument (IL, USA). Creatine kinase-MB (CK-MB) was assessed spectrophotometrically by a standard enzyme kit (Cat. No.1001055) bought from Spinreact, S.A.-Spain.

### Western blot analysis for HIF1-α, AKT, Smad-2, TGF-β & Bax & Bcl2

Liver, kidney and heart tissues were homogenized in buffer according to radio-immunoprecipitation assay (RIPA), which contains phosphatase and proteinase inhibitors. Bradford reagent was used to determine protein concentration [36]. 40 g proteins were transferred to 10% SDS/PAGE. Transferring of the separated proteins to nitrocellulose membranes occurred, and skimmed milk (5%) was used in tris-buffered saline/Tween-20 (TBST) for blocking. Then prodding the membranes with antibodies against HIF1-α, AKT, Smad-2, TGF-β and Bax and Bcl2 (Novus Biologicals, CO, USA) overnight at 4°C. Washing the blots with TBST and incubating them with the 2ry antibodies (1 h/room temperature). Protein bands were visualized using the ECL-Plus detection system (Amersham Life Sciences, Little Chalfont, Buckinghamshire, UK).

### Statistical analysis

Data were expressed as means ± SEM for quantitative measures. Statistical comparison between different groups were performed using one-way analysis of variance (ANOVA) followed by Tukey-Kramer multiple comparisons test using IBM SPSS Statistics program. Differences were considered significant at p < 0.05.

## Results

### Argi & Carn attenuate CoCl_2_-altered Hb concentration

The present study revealed that Hb concentration was significantly decreased in the CoCl_2_ -treated group compared with the normoxic group (p ≤ 0.001), while the administration of Carn and Argi either alone or in combination significantly increased Hb concentration with a mean value of 9.1, 8.5 and 9.07 respectively, compared with the CoCl_2_-treated group with the combination group revealing the most significant impact (Tables 1 & 2).

**Table 1.** List of different antibodies used.

HIF-1 alpha antibody (H1alpha67)
AKT1 [p Ser473] antibody (104A282) - BSA Free
Smad1 antibody (2A1)
TGF-beta antibody
Bax antibody
Bcl-2 antibody

**Table 2.** Hemoglobin concentration (g/dl) in controls as well as different treated groups.

Groups	Hb
Control	13.03± 0.77^a^
CoCl_2_	6.03± 0.13^b^
CoCl_2_ +Argi	9.1 ± 0.22^c^
CoCl_2_+ Carn	8.5 ±0.33^c^
CoCl_2_ +Argi +Carn	9.07± 0.35^c^

p < 0.05 is considered highly significant; Data were expressed as mean ± SEM; n=10. Different letters are significantly different from each other while similar letters are not significantly different.

### Argi & Carn attenuate CoCl_2_-induced liver injury

CoCl_2_-intoxicated rats demonstrated a significant elevation in serum ALT, and AST activities as illustrated in Table 3. Rats' pretreatment with Argi or/and Carn presented a significant improvement in the previously mentioned parameters with a mean value of 49.2, 50.98 And 46.77 respectively with the combination regimen reflecting the most significant impact.

**Table 3.** Markers of hepatic, renal and cardiac functions in the serum of rats.

Groups/parameter	Mean+ SE ALT activities (U/ld)	Mean+ SE AST activities (U/l)	Mean+ SE urea (mg/dl)	Mean+ SE creatinine (mg/dl)	Mean+ SE trop.T Pg/ml	Mean+ SE CK-MB U/l
Control	43.62 ± 0.9^a^	101.92 ±1.93^a^	23.8 ± 0.8^a^	0.81± 0.02^a^	38.6 ± 1.2^a^	29.12 ± 1.2^a^
CoCl_2_	81.84± 0.73 ^b^	147.7 ±1.02 ^b^	47.53 ± 0.67^b^	2.32 ± 0.1^b^	67.63 ± 1.2^b^	49.5 ± 1.5^b^
CoCl_2_ +Argi	49.2 ±1.1^a^	123.0 ±0.98^c^	32.6± 0.55^c^	1.23 ± 0.02^a^	47.47 ± 1.2^c^	40.75 ± 1.3^c^
CoCl_2_+ Carn	50.98 ±1.3^a^	125.3 ±1.05^c^	32.05 ± 0.65^c^	1.9 ± 0.23^b^	32.45 ± 1.7^a^	38.45 ± 1.2^c^
CoCl_2_ +Argi +Carn	46.77 ±0.99^a^	113.0 ±1.01^d^	29.7 ± 0.45^a^	1.18 ± 0.28^a^	43.23 ± 0.3^c^	30.2 ± 0.6^a^

Data are expressed as means ± SE (n = 10).

p ≤ 0.05, different letters are significantly different from each other while similar letters are not significantly different.

SE: standard error.

### Argi & Carn modulated CoCl_2_-Induced growth & apoptotic biomarkers

**Figure 1.**
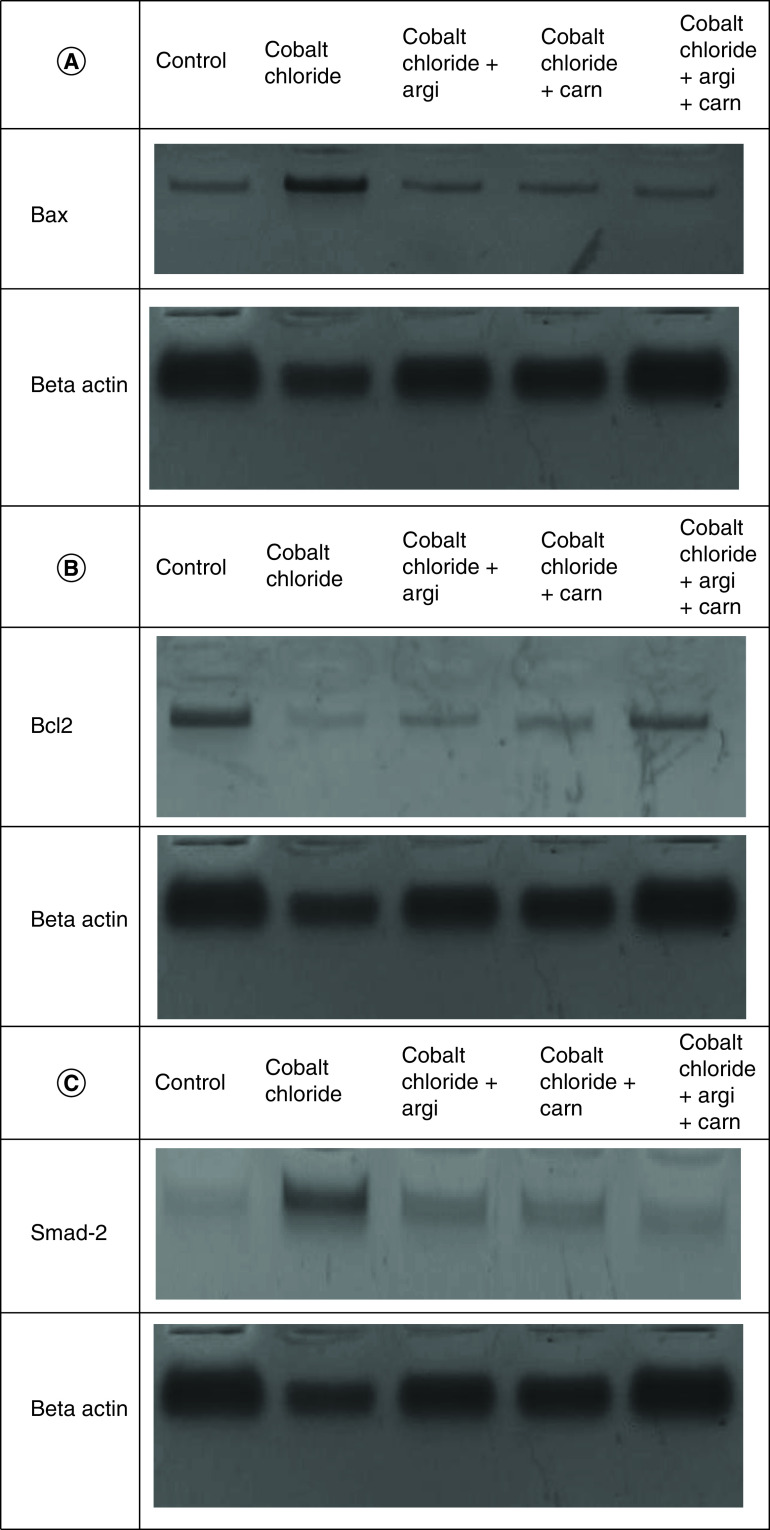
(A–C) Representative immunoblots (Western blot analysis) of hepatic Bax, Bcl 2, and Smad-2 in control and different treated groups.

**Figure 2.**
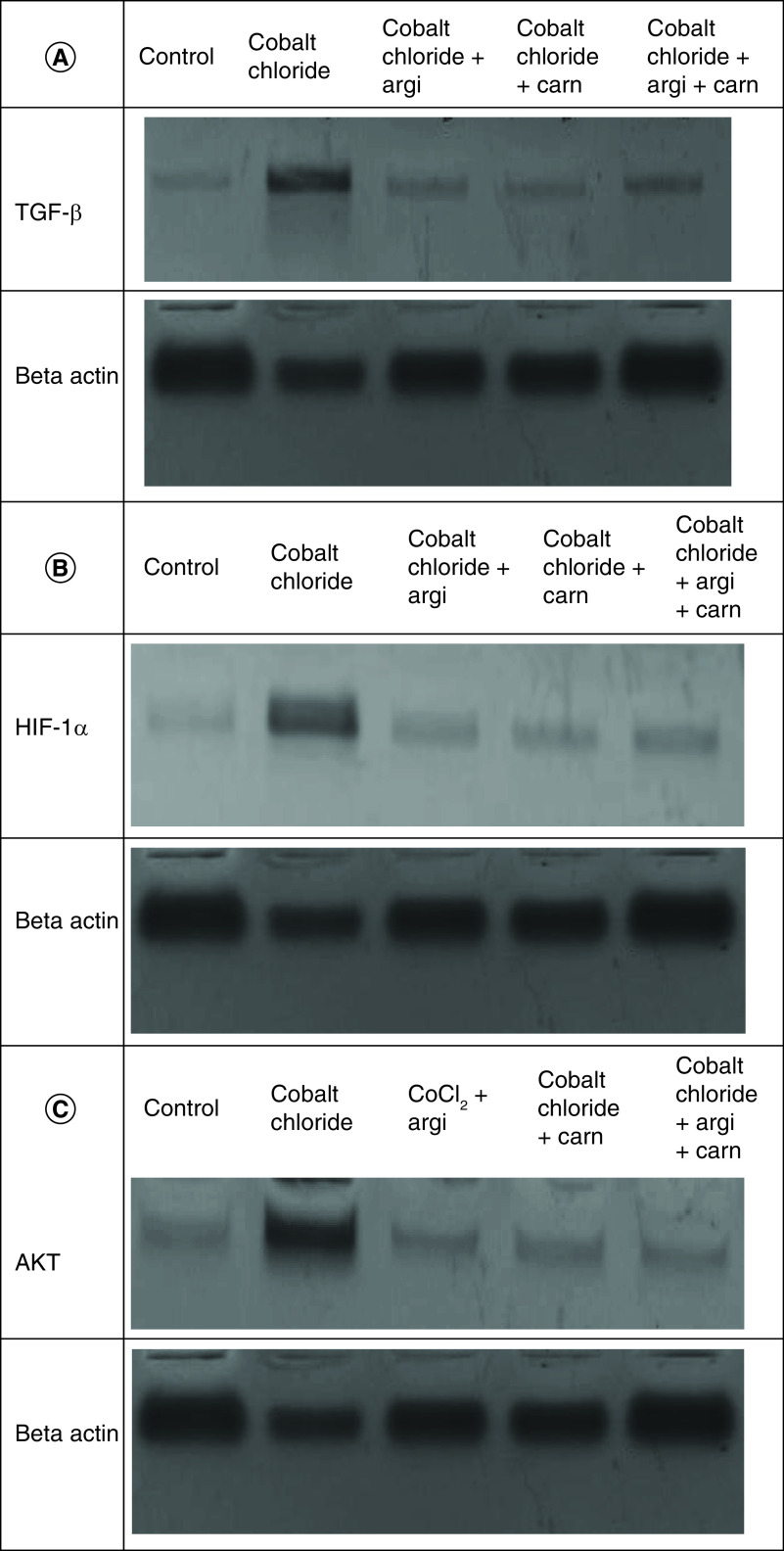
(A–C) Representative immunoblots (Western blot analysis) of hepatic TGF-β, HIF1-α, and AKT in control and different treated groups.

**Figure 3.**
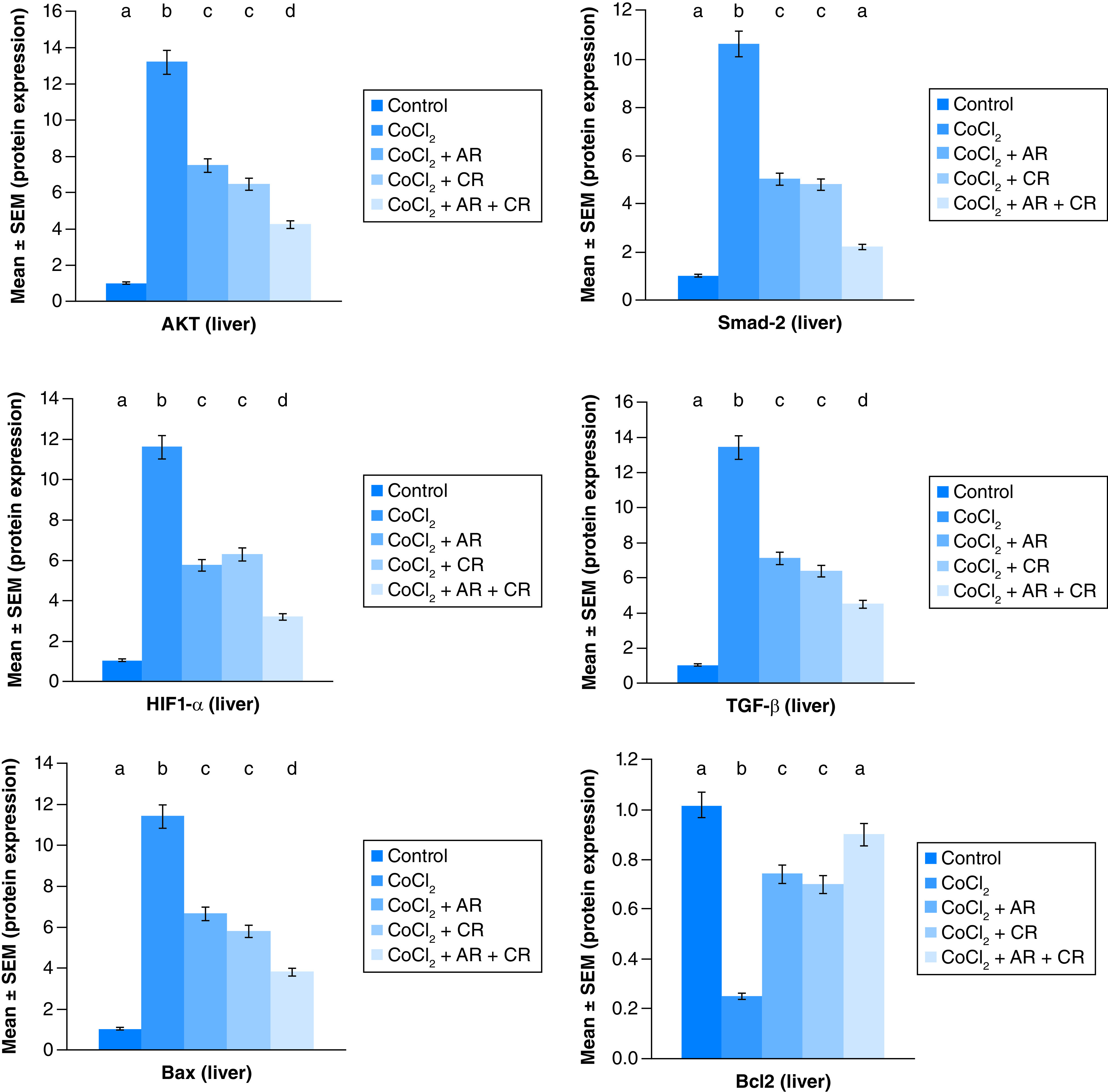
Impact of Arginine (AR), Carnosine (CR) and their combination on AKT, Smad-2, HIF1-α, TGF-β, Bax and Bcl2 protein expression post CoCl_2_ induced liver toxicity. Data are expressed as mean ± S.E.M (n = 10). p ≤ 0.05 value is considered significant. Groups having the same letter are not significantly different from each other, while those having different letters are significantly different from each other.

Figures 1–3 represent the immunoblots and quantitative analysis of hepatic Bax, Bcl 2, Smad-2 TGF-β, HIF1-α, and AKT protein expression, in control and different treated groups. western blot analysis results indicated that CoCl_2-_administration induced a highly significant elevation in HIF1-α, TGF-β, Smad-2, AKT and Bax protein expressions with a concomitant significant decrease in Bcl2 expression in hepatic tissue compared with the control value. Meanwhile, Carn and Argi administration alleviated the overexpression in HIF1-α, TGF-β, Smad-2, AKT and Bax, compared with the CoCl_2_-intoxicated group, while Bcl2 expression was significantly increased by the aforementioned treatments with the combination group revealing the most significant impact at P ≤ 0.05.

### Argi or/& Carn attenuate CoCl_2_-induced renal injury

In the current study, Table 3 pointed that CoCl_2_ induced an obvious increase in creatinine and urea levels indicating kidney dysfunction. However, antioxidants manipulation either solely or together depleted the aforementioned distorted biomarkers with a mean value of 1.23, 1.9 and 1.18 respectively for creatinine and 32.6, 32.05 and 29.7 respectively for urea compared with CoCl_2_ intoxicated group with the combination regimen illustrating the most significant impact.

**Figure 4.**
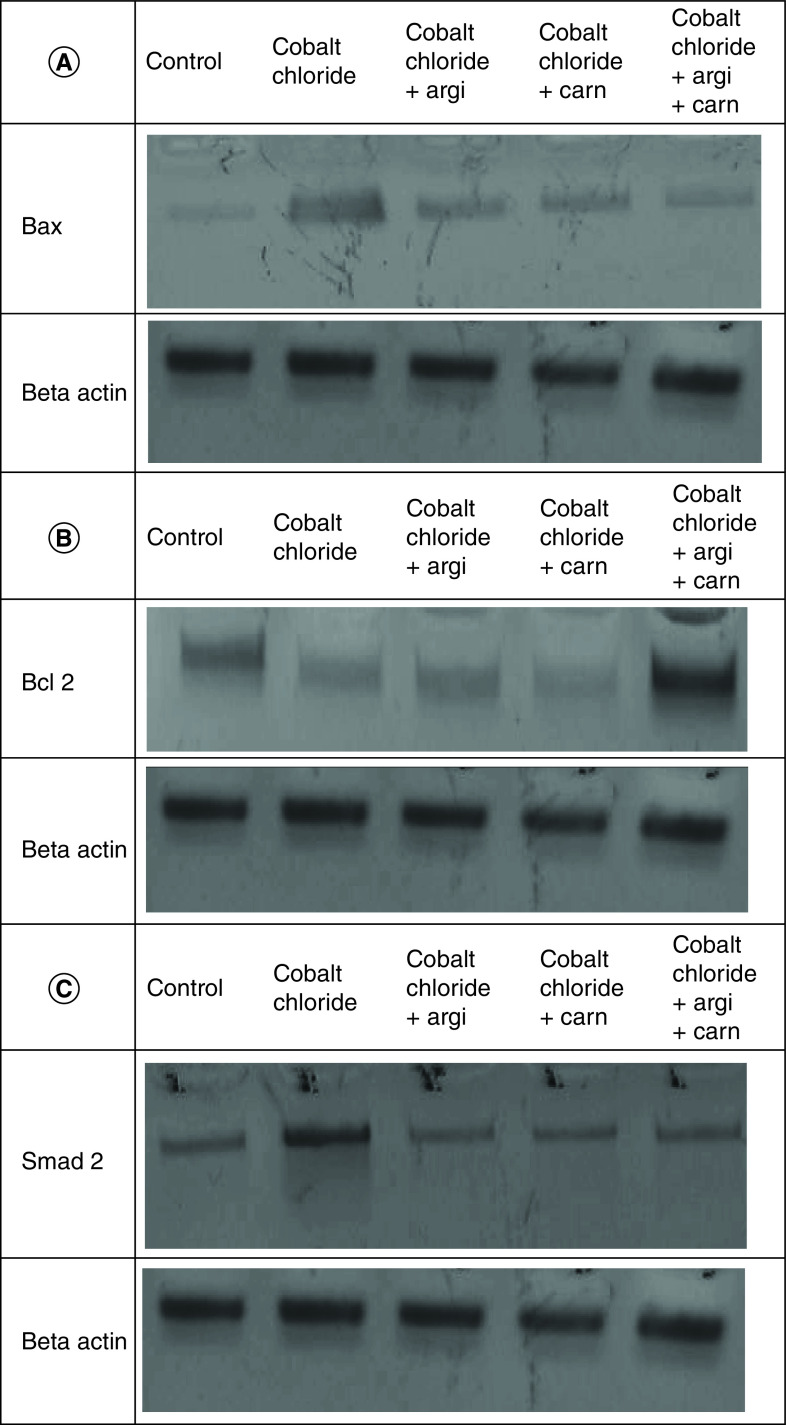
(A–C) Representative immunoblots (Western blot analysis) of renal Bax, Bcl 2, and Smad-2 in control and different treated groups.

**Figure 5.**
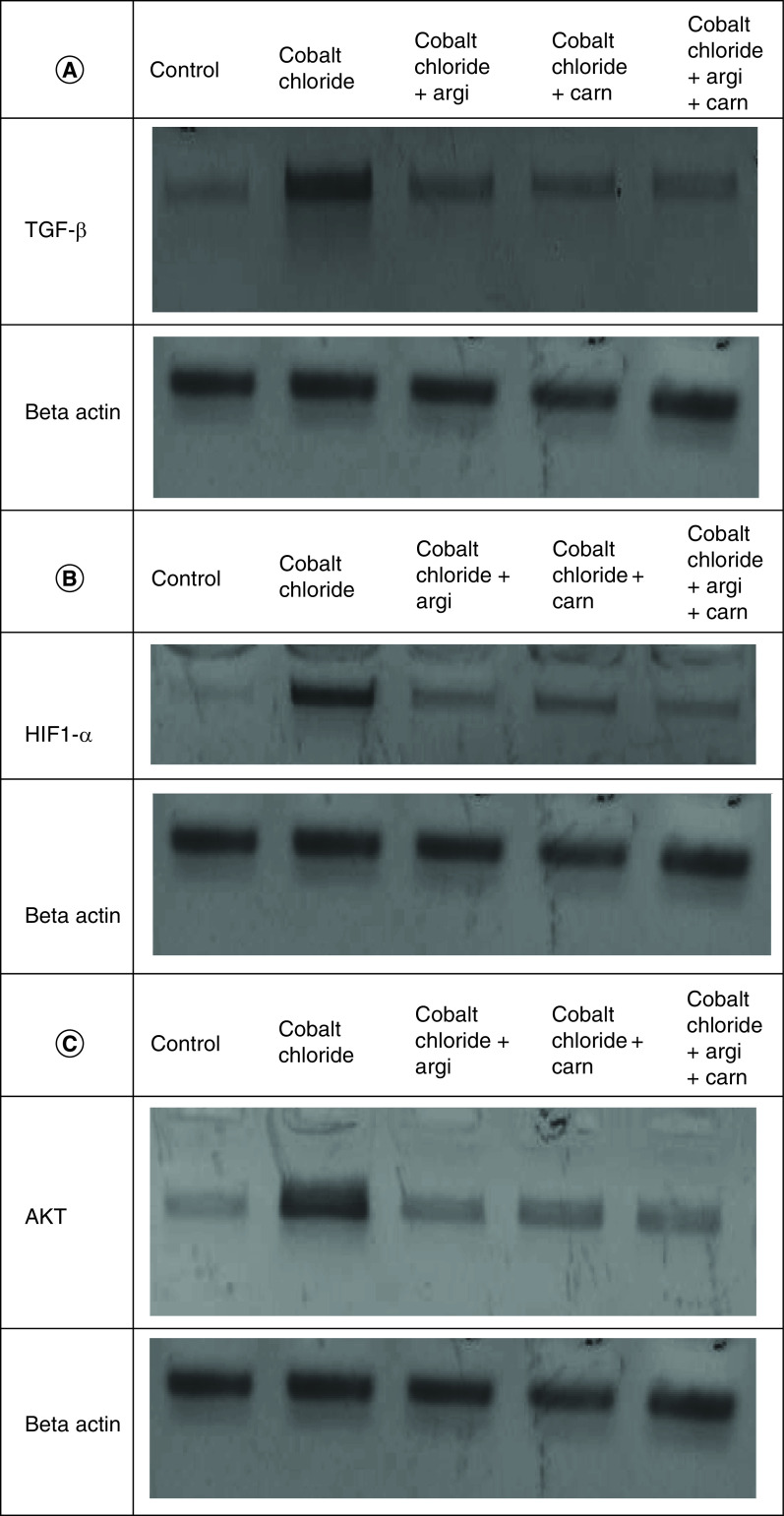
(A–C) Representative immunoblots (Western blot analysis) of renal TGF-β, HIF1-α, and AKT in control and different treated groups.

**Figure 6.**
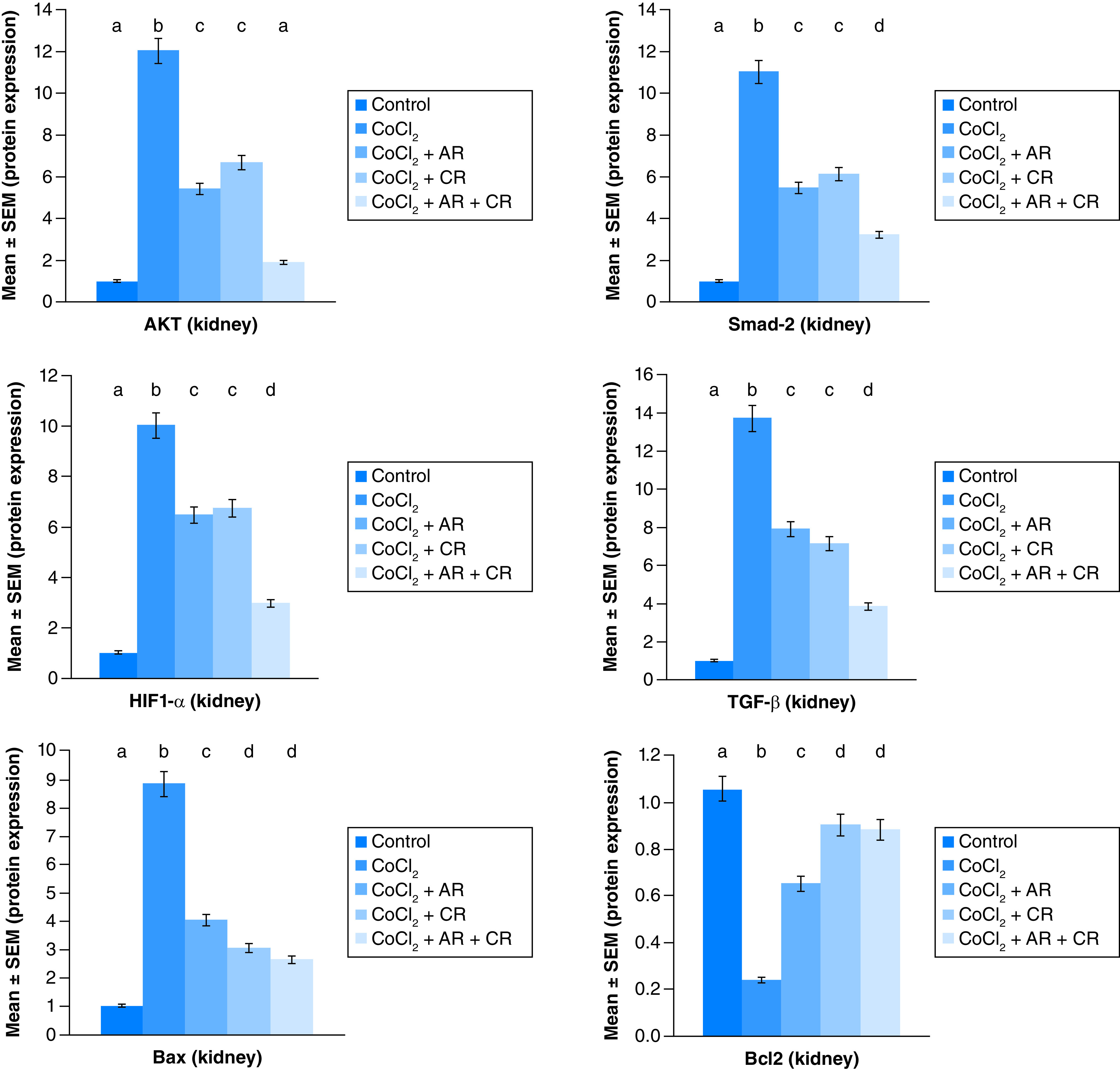
Impact of Arginine (AR), Carnosine (CR) and their combination on AKT, Smad-2, HIF1-α, TGF-β, Bax and Bcl2 protein expression post CoCl_2_ induced kidney toxicity. Data are expressed as mean ± S.E.M (n = 10). p ≤ 0.05 value is considered significant. Groups having the same letter are not significantly different from each other, while those having different letters are significantly different from each other.

The immunoblots and quantitative analysis of renal Bax, Bcl 2, Smad-2 TGF-β, HIF1-α, and AKT in control and different treated groups are presented in Figures 4–6.

CoCl_2-_administration promoted a significant rise in protein expressions of Bax, HIF1-α, TGF-β, Smad-2 and AKT accompanied with a significant depletion in Bcl2 expression in renal tissue as compared with a non-treated group (p ≤ 0.001). While antioxidants treatment improved Bax, HIF1-α, TGF-β, Smad-2, and AKT overexpression as compared with the group intoxicated with CoCl_2_, while such treatment significantly increased Bcl2 expression with the combination group revealing the most significant impact.

### Argi or/& Carn attenuate CoCl_2_-induced cardiac injury

Serum cardiac biomarkers including CK-MB and Trop- T in the control and other experimental groups intoxicated with CoCl_2_ are revealed in Table 3. The damage induced by CoCl_2_ significantly elevated these biomarkers with a mean value of 47.47, 32.45 and 43.23 respectively, for troponin and, 40.75, 38.45 and 30.2 respectively, for CK-MB while the use of antioxidants significantly ameliorated the deviation in the previous biomarkers, when compared with CoCl_2_-intoxicated group.

**Figure 7.**
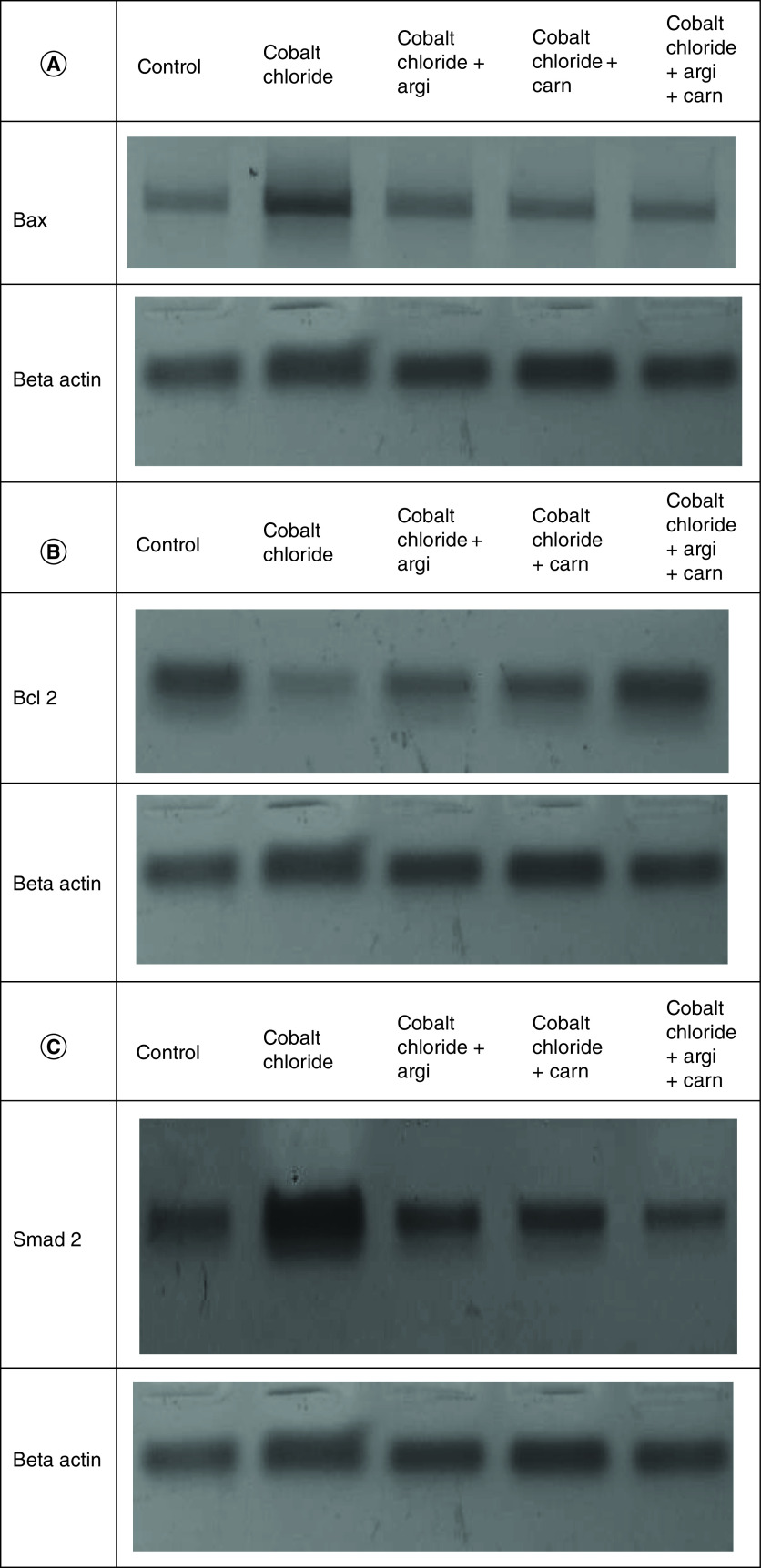
(A–C) Representative immunoblots (Western blot analysis) of cardiac Bax, Bcl 2, and Smad-2 in control and different treated groups.

**Figure 8.**
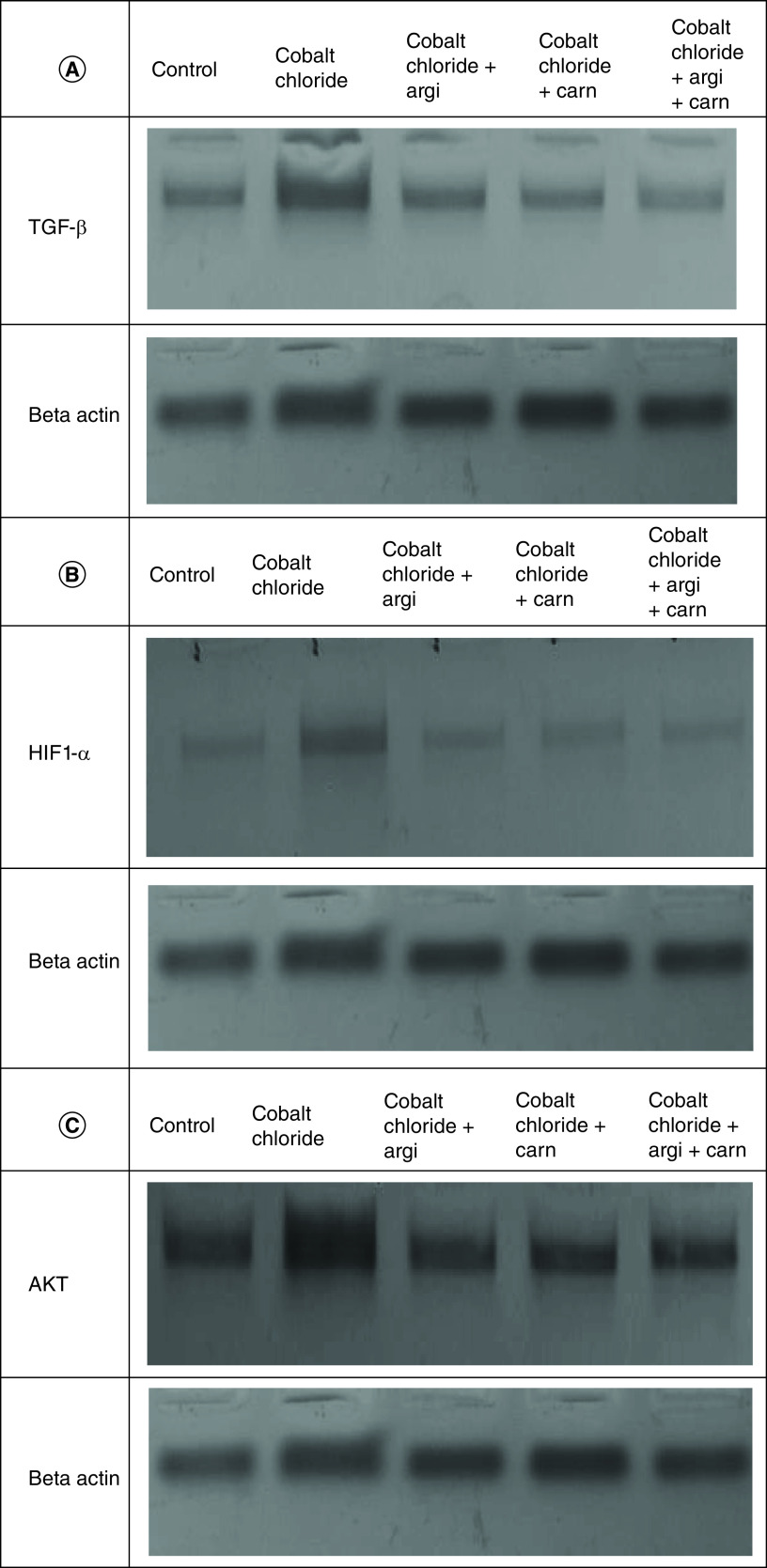
(A–C) Representative immunoblots (Western blot analysis) of cardiac TGF-β, HIF1-α, and AKT in control and different treated groups.

**Figure 9.**
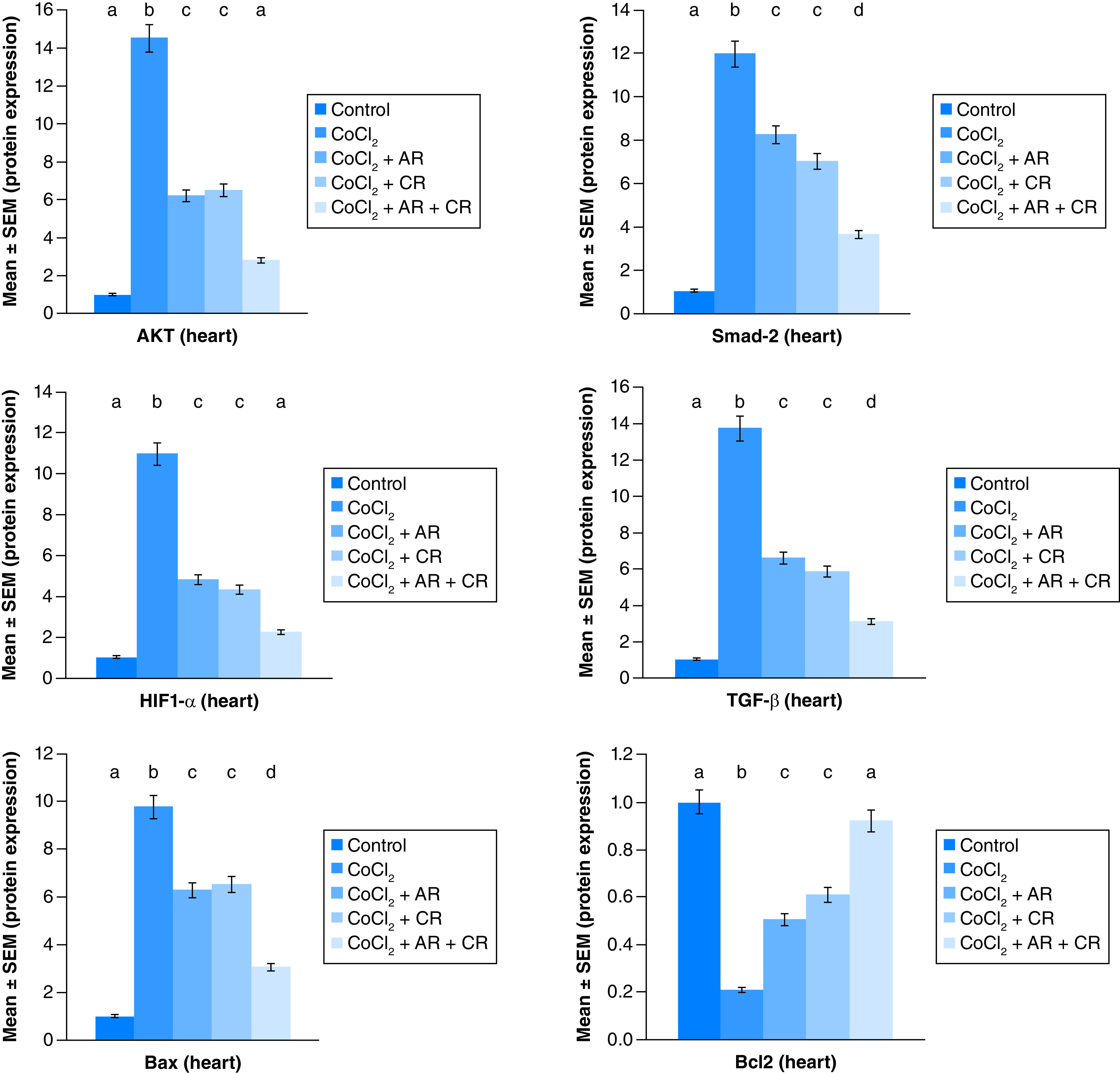
Impact of Arginine (AR), Carnosine (CR) and their combination on AKT, Smad-2, HIF1-α, TGF-β, Bax and Bcl2 protein expression post CoCl_2_ induced heart toxicity. Data are expressed as mean ± S.E.M (n = 10). p ≤ 0.05 value is considered significant. Groups having the same letter are not significantly different from each other, while those having different letters are significantly different from each other.

Figures 7–9 represent the immunoblots and quantitative analysis of cardiac Bax, Bcl 2, Smad-2 TGF-β, HIF1-α, and AKT, in control and different treated groups. Data of western blot analysis showed that CoCl_2-_intoxication illustrated a significant raise in Bax, HIF1-α, TGF-β, Smad-2, and AKT protein expressions, while it significantly decreased Bcl2 expression compared with normal value (p ≤ 0.001). Whereas, Carn and Argi treatment attenuated Bax, HIF1-α, TGF-β, Smad-2 and AKT over expression in cardiac tissue. Even though Bcl2 expression was significantly improved via these treatments with the combination treatment showing the most significant impact.

## Discussion

CoCl_2_ is a ubiquitous trace element that is widely distributed in the environment and utilized in the production of alloys, catalysts and diamonds. Nonetheless, it is categorized as a human health hazard. Excess dietary cobalt can impair various essential organs functions, which paves the way for understanding the toxicity of cobalt chloride in the liver, kidney and cardiac tissue. Previous evidence suggests that CoCl_2_-induced organ damage involves oxidative stress [37]. An imbalance between the antioxidant defense system and ROS levels brought on via excessive ROS production causes oxidative stress, inflammation, cell death, and DNA damage.

Herein, the administration of CoCl_2_ significantly increased the protein expressions of HIF-α. Meanwhile, a reduction in Hb concentration was significant. Nevertheless, Car and Arg significantly modulated these deviated parameters. According to some reports, CoCl_2_ generates oxygen-derived free radicals, which worsen the effects of oxidative stress. Moreover, cobalt salts have been shown to increase the expression of many stress-responsive proteins, including heme oxygenase [10,13]. It is believed that increased heme oxygenase activity may have significant antioxidant importance by enhancing the body's ability to defend against oxidative stress [38,39].

CoCl_2_ is a well-known chemical that mimics hypoxia; it causes the same effects in several aspects in various cultured cells, including loss of mitochondrial membrane potential, increase the release of ROS that causes cell death, and increased expression of HIF-1α and p53, which are implicated in the apoptotic pathway [15].

Erythropoietin transcription is activated by CoCl_2_ therapy in normoxia, and it has been proposed that cobaltous ions replace ferrous ions under heme, changing the shape of a heme protein O_2_ sensor [19]. HIF-1α is necessary for the induction of several genes during hypoxia, including erythropoietin and vascular endothelial growth factor. It causes Complex III's mitochondria to produce more ROS, which causes the HIF-1α protein to overexpress itself [48,49]. CoCl_2_ induces apoptosis by triggering the caspase-3 and p38 mitogen-activated protein kinase (MAPK) pathways [50]. L-carnosine was observed to decrease HIF-1a protein expression influencing its stability and reduce the HIF-1 transcriptional impact. L-carnosine is involved in ubiquitin-proteasome system promoting HIF-1a degradation.

In the current study, hepatic injury was evaluated by the measuring hepatic biomarkers (AST and ALT). Administration of CoCl_2_ induced a significant elevation in these enzymes' activity. These results coincide with other researches who revealed that CoCl_2_ exposure induced hepatic necrosis accompanied with rise in the activities of hepatic enzymes [40,41]. Hepatic damage is usually marked by the consequence release of AST and ALT [42]. The present data revealed that CoCl_2_ induced a noticeable elevation in creatinine and urea levels demonstrating renal dysfunction. CoCl_2_ induced renotoxicity was corroborated by an increase in serum blood urea nitrogen and creatinine. Recent studies discovered a positive link between kidney injury and elevated blood urea nitrogen and creatinine levels [43].

Serum CK-MB and Troponin were significantly elevated in the CoCl_2_ compared with normal group. Cobalt is present in the radical ions form in our body [44], it attacks cell membrane's unsaturated fatty acids and convert them into free radical (lipid and lipid peroxidation) [45]. Lipid peroxidation decreases fluidity of plasma membrane and elevates its fragility, with consequence impairing of membranous receptors and enzymes' function, affecting the membrane permeability, causing calcium ions' entry abnormalities, that causes destruction of the endoplasmic reticulum, and ends in cellular death [46]. Cellular death increase CK-MB, ALT and AST concentrations. It was reported that CoCl_2_ significantly increased CK-MB and LDH levels [47].

In rat liver, CoCl_2_ increases malondialdehyde, lowers glutathione (GSH), superoxide dismutase and catalase concentration. Alanine transaminase, aspartate aminotransferase, urea, creatinine and alkaline Phosphatase activity was elevated in CoCl_2_-induced hepatic and renal damage. Nonetheless, there was a decrease in lactate dehydrogenase activity [51]. The largest cobalt accumulation occurs in the liver. So, it is conceivable to hypothesize that cobalt could cause polyunsaturated fatty acid peroxidation, which would then cause phospholipid degradation and, ultimately, liver cell degeneration. When cobalt ions combine with ROS, they may produce cytotoxic hydroxyl radicals. The subsequent generation of free radicals by hydroxy radicals may lower cellular GSH levels, ascorbic acid concentrations and NADPH activity. Resultant ROS damages cellular proteins and DNA [52,53]. Novel experimental data suggest that the impact of hypoxia on renal cell survival, inflammatory cell recruitment and collagen gene expression may hasten the progression of chronic kidney disease.

The intoxication with CoCl_2_ in the current study profoundly increased the protein expression of AKT, Smad-2 and TGF-α in the liver, kidney and heart tissue.

Phosphoinositide 3-kinase (PI3K) and receptor tyrosine kinases control the activity of the essential enzyme Akt, which is necessary for cell survival (RTKs). CoCl_2_ enhances Akt phosphorylation in a manner that depends on both dose and time. The fact that Akt activation in response to CoCl_2_ was reduced when the PI3K/Akt pathway was blocked suggests that PI3K is required for the phosphorylation of Akt by CoCl_2_ [8,43,54].

Hepatic stellate cell (HSC) activation and fibrosis progression are two aspects of liver pathophysiology influenced by microRNA (miRNA)-mediated gene regulation. In the development of liver fibrosis, miR-942 expression was up regulated in activated HSCs and exhibited an inverse relationship with BAMBI expression. TGF-beta and lipopolysaccharide (LPS), two important factors in liver fibrosis and inflammation, cause HSCs to express miR-942 via Smad2/3 and NF-p50 binding to the miR-942 promoter, respectively. Mechanistically, the increased miR-942 degrades BAMBI mRNA in HSCs, making the cells more sensitive to fibrogenic TGF-signaling [55]. The relationship between active TGF and type II receptor (TGF-RII), which causes phosphorylated Smad2 and Smad3 to form a complex with Smad4 and migrate into the nucleus to regulate the expression of downstream genes, implicated in EMT [56].

Herein, the injection of CoCl_2_ dramatically raised Bax protein expressions. Bcl2 was dramatically reduced in the meantime. These aberrant apoptotic biomarkers were dramatically regulated by Car and Arg. CoCl_2_ increased oxidative stress, inflammation, and apoptosis. It has been documented that oxidative stress, inflammation, and apoptosis are connected to genotoxicity [57,58]. Yet, chronic inflammation brought on by persistent oxidative stress may be related to some chronic diseases, including cancer, diabetes, and cardiovascular conditions. The underlying etiology implicates the activation of various transcription factors such as NF-κB, p53, and NrF2 by oxidative stress ultimately leading to expressions of various genes including inflammatory cytokines, and chemokines [59,60].

Argi is an amino acid that plays an important role in immune function, wound healing, cell division, removing ammonia from the body, and hormone release. It is a precursor for nitric oxide (NO) synthesis, making it important in the regulation of blood pressure. Arginine is necessary for T-Cells to function in the body. It is known for its powerful antioxidant and anti-inflammatory power [21–23]. NO in its physiological values possess anti-apoptotic actions [24] and may promote healing during inflammation [61]. The small intestine and kidneys are the primary locations for arginine production.

Carn has important free radical scavenging ability, reduce lipid peroxidation, encourage the development of antioxidant enzymes and limit the production of pro-inflammatory cytokines [29,30]. It can protect cardiomyoblasts against HIF1-α induced hypoxia [31]. It can protect against ischemia/ reperfusion-induced acute renal failure in rats and lower rat mortality after cerebral ischemia [62].

### Study limitations

The number of animals and measured parameters caused some limitations to the experiment. Cobalt handling may cause asthma attacks with shortness of breath, coughing, wheezing, and chest tightness. It may affect the thyroid, heart, kidneys and liver. Repeated exposure can cause lungs fibrosis even if no signs are observed. Experiments on animals do not exactly mimic the way that the human body and diseases may respond to treatments, chemicals or drugs. Some compounds are highly toxic so small amounts may cause death. Future studies should investigate the effect of cobalt on different organs as the brain, testis and lung.

## Conclusion

Intriguingly, the combined treatment with both Carn and Argi therapy was regarded as a potential candidate for halting the course of multi-organ dysfunction brought on by CoCl_2_ via reversing the altered protein expression of growth factors and apoptotic biomarkers including HIF1-α, AKT, Smad-2, TGF-, Bax, and Bcl2 produced by CoCl_2_ in the liver, kidney, and heart.

**Summary points**

- Induction of cardiac, hepatic and renal dysfunction via CoCl_2_ in rats.
- Pretreatment with Arginine, Carnosine and their combination for one month.
- Monitoring the protective impact of Arginine and Carnosine.
- Monitoring AKT, Smad-2, TGF-β, Bax/Bcl2 ratio, HIF-α protein expression in heart, liver and kidney.

## References

[1] Ajibade AJ, Ogunmola IA, Okeleye AE. Ameliorative Effects of Moringa oleifera Leaf Extract on the Cobalt Chloride-Induced Liver Damage in Adult Wistar Rats. Sys. Rev. Pharm. 12(12), 3865–3871 (2021).

[2] Karakka Kal AK, Perwad Z, Karatt TK, Nalakath J, Subhahar M. Using inductively coupled plasma mass spectrometry to assess essential and performance-enhancing metals in the urine of racehorses. J. Anal. Toxicol. 44(5), 490–498 (2020). 32020176 10.1093/jat/bkaa004

[3] Chen RJ, Lee VR. Cobalt Toxicity. In: StatPearls. StatPearls Publishing, FL, USA (2022). PMID: 36508548. 36508548

[4] Mohamed AA, Metwally MM, Khalil SR, Salem GA, Ali HA. Moringa oleifera extract attenuates the CoCl2 induced hypoxia of rat's brain: expression pattern of HIF-1α, NF-kB, MAO and EPO. Biomedicine & Pharmacotherapy. 109, 1688–1697 (2019). 30551423 10.1016/j.biopha.2018.11.019

[5] Oyagbemi AA, Ajibade TO, Esan OO Naringin abrogates angiotensin-converting enzyme (ACE) activity and podocin signaling pathway in cobalt chloride-induced nephrotoxicity and hypertension. Biomarkers. 28(2), 206–216 (2022). 36480283 10.1080/1354750X.2022.2157489

[6] Oyagbemi AA, Akinrinde AS, Adebiyi OE Luteolin supplementation ameliorates cobalt-induced oxidative stress and inflammation by suppressing NF-кB/Kim-1 signaling in the heart and kidney of rats. Env. Toxicol. Pharmacol. 80, 103488 (2020). 10.1016/j.etap.2020.103488 32898663

[7] Tong Y, Tong K, Zhu Q Cobalt chloride induced apoptosis by inhibiting GPC3 expression via the HIF-1α/c-Myc axis in HepG2 cells. OncoTargets and therapy. 12, 10663–70 (2019). 31824173 10.2147/OTT.S227215 PMC6901039

[8] Lee YW, Cherng YG, Yang ST, Liu SH, Chen TL, Chen RM. Hypoxia induced by cobalt chloride triggers autophagic apoptosis of human and mouse drug-resistant glioblastoma cells through targeting the PI3K-Akt-mTOR signaling pathway. Oxid. Med. Cell. Long. 2021, 1–6 (2021). 10.1155/2021/5558618 PMC8177987 34136065

[9] Oria RS, Obeten KE, Etetim AE, Mgbolu PE, Ikoku GI, Ijomone OM. Ameliorative effect of prosopis africana seed extract on cobalt chloride induced cerebellar toxicity: neurobehavioural, histomorphological and biochemical findings. Niger. J. Neurosci. 13(1), 9–19 (2022).

[10] European Chemicals Agency. ECHA Committee for Risk Assessment (RAC) Background Document to the Opinion Proposing Harmonised Classification and Labelling at EU Level of Cobalt. CLH-O-0000001412-86-172/F, Helsinki, Finland (2017).

[11] Kirkland D, Brock T, Haddouk H, Hargeaves V, Lloyd M, McGarry S New investigations into the genotoxicity of cobalt compounds and their impact on overall assessment of genotoxic risk Regul. Toxicol. Pharmacol. 73 (1), 311–338 (2015). 10.1016/j.yrtph.2015.07.016 26210821

[12] NTP Toxicology Studies of Cobalt Metal (CAS No. 7440-48-4) in F344/N Rats and B6C3F1/N Mice and Toxicology and Carcinogenesis Studies of Cobalt Metal in F344/NTac Rats and B6C3F1/N Mice (Inhalation Studies). In: National Toxicology Program Technical Report Series. National Toxicology Program Technical Report Series No. 581 US Department of Health and Human Services, WA, USA (2014).

[13] European Chemicals Agency. ECHA Committee for Risk Assessment (RAC) Committee for Socio-economic Analysis (SEAC) Opinion on an Annex XV dossier proposing restrictions on: Cobalt sulphate, Cobalt dichloride, Cobalt dinitrate, Cobalt carbonate and Cobalt di(acetate). In: European Chemicals Agency; Opinion of the Committee for Risk Assessment and Opinion of the Committee for Socio-economic Analysis. ECHA/RAC/RES-O-0000006741-74-01/F, Helsinki, Finland (2020).

[14] Taxell P, Pasi H. Toxicity assessment and health hazard classification of stainless steels. Reg. Toxicol. Pharmacol. 133, 105227 (2022). 10.1016/j.yrtph.2022.105227 35817207

[15] Lan A, Liang-Can X, Yang LZ, Chun-Tao Y, Xiu-Yu W, Pei-Xxi C Interaction between ROS and p38MAPK contributes to chemical hypoxia-induced injuries in PC12 cells. Mol. Med. Rep. 5(1), 250–255 (2012). 21993612 10.3892/mmr.2011.623

[16] Wood JG, Johnson JS, Mattioli LF, Gonzalez NC. Systemic hypoxia promotes leukocyte-endothelial adherence via reactive oxidant generation. J. Appl. Physiol. 87, 1734–1740 (1999). 10562616 10.1152/jappl.1999.87.5.1734

[17] Kong D, Zhang F, Shao J, Wu L, Zhang X, Chen L Curcumin inhibits cobalt chloride-induced epithelial-to-mesenchymal transition associated with interference with TGF-β/Smad signaling in hepatocytes. Lab. Invest. 95(11), 1234–1245 (2015). 26302188 10.1038/labinvest.2015.107

[18] Petrova E, Pavlova E, Tinkov AA Cobalt accumulation and iron-regulatory protein profile expression in immature mouse brain after perinatal exposure to cobalt chloride. Chemico-Biolog. Inter. 329, 109217 (2020). 10.1016/j.cbi.2020.109217 32750324

[19] Paneerselvam C, Ganapasam S. β-Escin alleviates cobalt chloride-induced hypoxia-mediated apoptotic resistance and invasion via ROS-dependent HIF-1α/TGF-β/MMPs in A549 cells. Toxicol. Res. 9(3), 191–201 (2020). 10.1093/toxres/tfaa019 PMC7329168 32670550

[20] Li W, Sun K, Hu F Protective effects of natural compounds against oxidative stress in ischemic diseases and cancers via activating the Nrf2 signaling pathway: a mini review. J. Biochem. Mol. Toxicol. 35(3), e22658 (2021). 33118292 10.1002/jbt.22658

[21] Tapiero H, Mathe G, Couvreur P, Tew KD. I. arginine. Biomedicine & pharmacotherapy. 56(9), 439–445 (2002). 12481980 10.1016/s0753-3322(02)00284-6

[22] Chen SF, Pan MX, Tang JC Arginine is neuroprotective through suppressing HIF-1α/LDHA-mediated inflammatory response after cerebral ischemia/reperfusion injury. Mol. brain. 13(1), 1–3 (2020). 32321555 10.1186/s13041-020-00601-9 PMC7175589

[23] Pérez de la Lastra JM, Curieses Andrés CM, Andrés Juan C, Plou FJ, Pérez-Lebeña E. Hydroxytyrosol and Arginine as Antioxidant, Anti-Inflammatory and Immunostimulant Dietary Supplements for COVID-19 and Long COVID. Foods. 12(10), 1937 (2023). 37238755 10.3390/foods12101937 PMC10217518

[24] Gorabi AM, Kiaie N, Hajighasemi S Statin-induced nitric oxide signaling: mechanisms and therapeutic implications. J. clin. med. 8(12), 2051 (2019). 31766595 10.3390/jcm8122051 PMC6947613

[25] Bailey JD, Diotallevi M, Nicol T Nitric oxide modulates metabolic remodeling in inflammatory macrophages through TCA cycle regulation and itaconate accumulation. Cell reports. 28(1), 218–230 (2019). 31269442 10.1016/j.celrep.2019.06.018 PMC6616861

[26] El Fakahany GA, Nassar SAEA, Elballat SES. Alterations in Kidney of Albino Rat due to Acrylamide Exposure and the Possible Protective Role of l-arginine (Biochemical, Histological, Immunohistochemical and Molecular Study). Sys. Rev. Pharm. 12(3), 744–752 (2021).

[27] Soliman N, El_Beltagy MA, Abdelrazek HM, Gouda SG. Histological and biochemical study of the protective effects of Uncaria tomentosa versus L-arginine against fipronil-induced nephrotoxicity in male albino rats. Egypt. J. Histol. 46(1), 460–477 (2023).

[28] Agrawal A, Rathor R, Kumar R, Singh SN, Kumar B, Suryakumar G. Endogenous dipeptide-carnosine supplementation ameliorates hypobaric hypoxia-induced skeletal muscle loss via attenuating endoplasmic reticulum stress response and maintaining proteostasis. IUBMB life. 74(1), 101–116 (2022). 34455667 10.1002/iub.2539

[29] Owoyele BV, Bakare AO, Olaseinde OF Synergistic interaction between acetaminophen and L-carnosine improved neuropathic pain via NF-κB pathway and antioxidant properties in chronic constriction injury model. The Korean J. Pain. 35(3), 271–279 (2022). 35768982 10.3344/kjp.2022.35.3.271 PMC9251391

[30] Aldini G, de Courten B, Regazzoni L Understanding the antioxidant and carbonyl sequestering activity of carnosine: direct and indirect mechanisms. Free Rad. Res. 55(4), 321–330 (2021). 10.1080/10715762.2020.1856830 33302741

[31] Feehan J, Hariharan R, Buckenham T Carnosine as a potential therapeutic for the management of peripheral vascular disease. Nut. Metabol. Cardiovas. Dis. 32(10), 2289–2296 (2022). 10.1016/j.numecd.2022.07.006 35973888

[32] Sandner P, Wolf K, Bergmaier U, Gess B, Kurtz A. Induction of VEGF and VEGF receptor gene expression by hypoxia: divergent regulation *in vivo* and *in vitro*. Kidney Int. 51(2), 448–453 (1997). 9027720 10.1038/ki.1997.60

[33] Al-Rasheed NM, Fadda LM, Al-Rasheed NM, Attia H, Ali HM, El-Agami H. Role of natural antioxidants in the modulation of plasma amino acid pattern in rats exposed to hemic hypoxia. Braz. Arch. Biol. Technol. 58(5), 741–749 (2015).

[34] Ali SA, Aly HF, Faddah LM, Zaidi ZF. Dietary supplementation of some antioxidants against hypoxia. World J. Gastroenterol. 18(44), 6379–6386 (2012). 23197883 10.3748/wjg.v18.i44.6379 PMC3508632

[35] Kjeldsberg CR. Principles of hematologic examination. In: Wintrobe's clinical hematology. Lee GR, Bittell TC, Foerster Jet al. (Eds). Philadelphia, London, 1, 7–37 (1993).

[36] Bradford MM. A rapid and sensitive method for the quantitation of microgram quantities of protein utilizing the principle of protein-dye binding. Anal. Biochem. 72, 248–254 (1976). 942051 10.1016/0003-2697(76)90527-3

[37] Garoui E, Troudi A, Fetoui H, Soudani N, Boudawara T, Zeghal N. Propolis attenuates cobalt induced nephrotoxicity in adult rats and their progeny. Exp. Toxicol. Pathol. 64, 837–846 (2012). 21507616 10.1016/j.etp.2011.03.004

[38] Qiong W, Wen-Shuang W, Lin S Mitochondrial Ferritin is a HIF1α-Inducible Gene that Protects from Hypoxia-Induced Cell Death in Brain. Antioxid. redox signal. 30(2), 198–212 (2019). 29402144 10.1089/ars.2017.7063

[39] Liao X, Zhang Z, Ming M, Zhong S, Chen J, Huang Y. Imperatorin exerts antioxidant effects in vascular dementia via the Nrf2 signaling pathway. Scient. Rep. 13(1), 5595 (2023). 10.1038/s41598-022-21298-x PMC10076271 37019901

[40] Okerenta OBM, Anacletus FC. Hepatoprotective and ameliorative effects of selected antioxidants on aluminium induced toxicity in wistar rats. European Journal of Adv. Res. Bio. Life Sci. 4(2), 24–34 (2016).

[41] Yakubu OE, Nwodo OFC, Imo C, Abdulrahman M, Uyeh LB. Effects of vitex doniana leaf extract on aluminium induced toxicity in male albino wistar rats. J. Appl. Biol. Biotechnol. 4(5), 37–40 (2016).

[42] Naik P. edn.) Biochemistry (3rd Edition) Jaypee Publishers, New Delhi, INDIA, 564–565 (2010).

[43] Iji OT, Ajibade TO, Esan OO Ameliorative effects of glycine on cobalt chloride-induced hepato-renal toxicity in rats. Animal Models Exp. Med. 6(2), 168–177 (2023). 10.1002/ame2.12315 PMC10158950 37141004

[44] Czarnek K, Terpiłowska S, Siwicki AK. Selected aspects of the action of cobalt ions in the human body. Cent. Eur. J. Immunol. 40(2), 236–242 (2015). 26557039 10.5114/ceji.2015.52837 PMC4637398

[45] Misra M, Rodriguez RE, Kasprzak KS. Nickel induced lipid peroxidation in the rat: correlation with nickel effect on antioxidant defense systems. Toxicol. 64, 1–17 (1990). 10.1016/0300-483x(90)90095-x 1977209

[46] Yaman SO, Ayhanci A. Lipid peroxidation. Accent. Lipid Peroxid. 12, 1–1 (2021).

[47] Liu M, Liu P, Zheng B Cardioprotective effects of alantolactone on isoproterenol-induced card. Pharmacol. 36, 20587384211051993 (2022). 10.1177/20587384211051993 PMC8744082 34986670

[48] Chandel NS, McClintock DS, Feliciano CE, Wood TM, Andres Melendez J, Rodriguez AM Hepatotoxicity of Cobalt Chloride in Albino Rat. Pharmacologyonline. 2, 179–185 (2011).

[49] Oyagbemi AA, Omobowale TO, Awoyomi OV Cobalt chloride toxicity elicited hypertension and cardiac complication via induction of oxidative stress and upregulation of COX-2/Bax signaling pathway. Hum. Exp. Toxicol. 38(5), 519–532 (2019). 30596275 10.1177/0960327118812158

[50] Schönberger T, Fandrey J, Prost-Fingerle K. Ways into understanding HIF inhibition. Cancers. 13(1), 159 (2021). 33466454 10.3390/cancers13010159 PMC7796500

[51] Basavaraju AM, Shivanna N, Yadavalli C, Garlapati PK, Raghavan AK. Ameliorative effect of Ananas comosus on cobalt chloride-induced hypoxia in Caco2 cells via HIF-1α, GLUT 1, VEGF, ANG and FGF. Biolo. trace element res. 199, 1345–1355 (2021). 10.1007/s12011-020-02278-6 32654099

[52] Wang Z, Zhao G, Zibrila AI, Li Y, Liu J, Feng W. Acetylcholine ameliorated hypoxia-induced oxidative stress and apoptosis in trophoblast cells via p38 MAPK/NF-κB pathway. Mol. Hum. Reprod. 27(8), gaab045 (2021). 34245298 10.1093/molehr/gaab045

[53] Lugun O, Singh J, Thakur RS, Pandey AK. Cobalt oxide (Co3O4) nanoparticles induced genotoxicity in Chinese hamster lung fibroblast (V79) cells through modulation of reactive oxygen species. Mutagen. 37(1), 44–59 (2022). 10.1093/mutage/geac005 35230445

[54] Liu J, Kong L, Chen D Bilirubin oxidation end product B prevents CoCl2-induced primary cortical neuron apoptosis by promoting cell survival Akt/mTOR/p70S6K signaling pathway. Biochem. Biophys. Res. Comm. 602, 27–34 (2022). 35247701 10.1016/j.bbrc.2022.02.063

[55] Hou Y, Zhang Y, Jiang S Salidroside intensifies mitochondrial function of CoCl2-damaged HT22 cells by stimulating PI3K-AKT-MAPK signaling pathway. Phytomed. 109, 154568 (2023). 10.1016/j.phymed.2022.154568 36610162

[56] Li H, Liu T, Yang Y Interplays of liver fibrosis-associated microRNAs: molecular mechanisms and implications in diagnosis and therapy. Genes & Diseases 10(4), 1457–1469 (2022). 37397560 10.1016/j.gendis.2022.08.013 PMC10311052

[57] Zheng JH, Viacava Follis A, Kriwacki RW, Moldoveanu T. Discoveries and controversies in BCL-2 protein-mediated apoptosis. Febs J. 283, 2690–2700 (2016). 26411300 10.1111/febs.13527

[58] El-Shorbagy HM, Eissa SM, Sabet S, El-Ghor AA. Apoptosis and oxidative stress as relevant mechanisms of antitumor activity and genotoxicity of ZnO-NPs alone and in combination with N-acetyl cysteine in tumor-bearing mice. Inter. J. Nanomed. 3911–3928 (2019). 10.2147/IJN.S204757 PMC6549730 31213808

[59] Strasen J, Sarma U, Jentsch M Cell-specific responses to the cytokine TGFβ are determined by variability in protein levels. Mol. Syst. Biol. 14(1), e7733–17 (2018). 29371237 10.15252/msb.20177733 PMC5787704

[60] Nosi D, Lana D, Giovannini MG, Delfino G, Zecchi-Orlandini S. Neuroinflammation: integrated nervous tissue response through intercellular interactions at the “whole system” scale. Cells. 10, 1195 (2021). 34068375 10.3390/cells10051195 PMC8153304

[61] Qin C, Yang S, Chu YH Signaling pathways involved in ischemic stroke: molecular mechanisms and therapeutic interventions. Signal Transd. Targeted Ther. 7(1), 215 (2022). 10.1038/s41392-022-01064-1 PMC9259607 35794095

[62] Zeb A, Cha JH, Noh AR Neuroprotective effects of carnosine-loaded elastic liposomes in cerebral ischemia rat model. J. Pharmac. Inves. 50, 373–381 (2020).

